# Suppression of progranulin expression inhibits bladder cancer growth and sensitizes cancer cells to cisplatin

**DOI:** 10.18632/oncotarget.9556

**Published:** 2016-05-23

**Authors:** Simone Buraschi, Shi-Qiong Xu, Manuela Stefanello, Igor Moskalev, Alaide Morcavallo, Marco Genua, Ryuta Tanimoto, Ruth Birbe, Stephen C. Peiper, Leonard G. Gomella, Antonino Belfiore, Peter C. Black, Renato V. Iozzo, Andrea Morrione

**Affiliations:** ^1^ Department of Pathology, Anatomy and Cell Biology and The Cancer Cell Biology and Signaling Program, Kimmel Cancer Center, Thomas Jefferson University, PA, Philadelphia, USA; ^2^ Department of Urology and Biology and The Prostate Cancer Program, Kimmel Cancer Center, Thomas Jefferson University, PA, Philadelphia, USA; ^3^ Vancouver Prostate Centre, Department of Urologic Sciences, University of British Columbia, Vancouver, Canada; ^4^ Department of Health and Endocrinology, University Magna Graecia of Catanzaro, Catanzaro, Italy

**Keywords:** progranulin, bladder cancer, motility, anchorage-independent growth, tumor formation in vivo

## Abstract

We have recently demonstrated a critical role for progranulin in bladder cancer. Progranulin contributes, as an autocrine growth factor, to the transformed phenotype by modulating Akt-and MAPK-driven motility, invasion and anchorage-independent growth. Progranulin also induces F-actin remodeling by interacting with the F-actin binding protein drebrin. In addition, progranulin is overexpressed in invasive bladder cancer compared to normal tissue controls, suggesting that progranulin might play a key role in driving the transition to the invasive phenotype of urothelial cancer. However, it is not established whether targeting progranulin could have therapeutic effects on bladder cancer. In this study, we stably depleted urothelial cancer cells of endogenous progranulin by shRNA approaches and determined that progranulin depletion severely inhibited the ability of tumorigenic urothelial cancer cells to migrate, invade and grow in anchorage-independency. We further demonstrate that progranulin expression is critical for tumor growth *in vivo*, in both xenograft and orthotopic tumor models. Notably, progranulin levels correlated with response to cisplatin treatment and were upregulated in bladder tumors. Our data indicate that progranulin may constitute a novel target for therapeutic intervention in bladder tumors. In addition, progranulin may serve as a novel biomarker for bladder cancer.

## INTRODUCTION

Bladder cancer is a major epidemiological issue in the United States with 74,000 estimated new cases and 16,000 estimated deaths in 2015 [1]. About 70% of early stages bladder tumors present as low-grade noninvasive papillary tumors (Ta stage). The remaining comprises tumors that have penetrated the basement membrane but not invaded the muscle layer of the bladder wall (T1 stage) and muscle-invasive tumors (T2-T4 stages) [2]. While the prognosis for low-grade tumors is generally good, about 15% of these patients develop invasive disease. For invasive tumors the prognosis is much less favorable, with only 50% survival at 5 years [3]. Invasive tumors are frequently associated with metastasis, which is associated with a 5 year survival rate of 6% [3]. Compared to other cancers, bladder cancer has the highest cost per patients in the US due to disease prevalence and costs associated with long-term monitoring [4]. However, despite the significant clinical impact of bladder cancer, there is still an urgent need to discover novel predictor of disease progression and novel therapeutic targets.

Progranulin, also known as proepithelin, PCDGF (PC-derived growth factor), granulin-epithelin precursor or acrogranin, is an evolutionary-conserved, secreted glycoprotein containing 7 and a half granulin (GRN) repeats which plays an important role as a *bona fide* growth factor in cell proliferation, angiogenesis, wound healing and transformation in several cancer systems [5–7]. Progranulin binds to the basement membranes of endothelial cells via a direct physical interaction with perlecan [8], an heparan sulfate proteoglycan affecting various biological functions including tumor angiogenesis [9–12].

In addition, progranulin regulates inflammation and neurodegeneration [13], and has been causatively linked to the development of frontotemporal dementia (FTD).

We have recently established that progranulin plays a critical role in bladder cancer progression [14, 15]. Progranulin promotes motility and invasion of urothelial cancer cells through the activation of the Akt and MAPK pathways and MAPK-dependent activation of paxillin, which may regulate focal adhesion dynamics [14, 15]. In addition, progranulin regulates F-actin remodeling by interacting with the F-actin binding protein drebrin [16–18], which is critical for progranulin-dependent urothelial cancer cell motility and invasion [18]. Importantly, drebrin regulates tumor growth *in vivo* [18] and its expression levels correlate with bladder tumor progression [18]. Indeed, progranulin expression is upregulated in invasive bladder cancer tissues vis-à-vis non-neoplastic tissues and it is detectable in the urine [15]. Thus, progranulin may be critical for the transition to the invasive phenotype of bladder cancer and may serve as a novel biomarker for bladder cancer.

In spite of this emerging body of evidence pointing to a critical role of progranulin in bladder cancer, it is not yet established whether targeting progranulin could affect tumorigenicity of urothelial cancer cells. Here we show that stable progranulin depletion using small hairpin RNA (shRNA) interference severely inhibited motility, invasion and anchorage-independent growth of tumorigenic UMUC-3 and T24T urothelial carcinoma-derived cells. In addition, progranulin targeting markedly reduced *in vivo* tumor growth of UMUC-3 cells in both orthoptopic and subcutaneous xenograft tumor models. Importantly, progranulin depletion sensitized urothelial cancer cells to cisplatin treatment, further proving a pro-survival function of progranulin. Finally, enhanced progranulin expression in a bladder cancer tissue microarray correlated with tumorigenicity. Collectively, these results suggest that progranulin may work as a novel therapeutic target for bladder cancer and could serve as novel biomarker for bladder cancer.

## RESULTS

### Progranulin depletion inhibits motility of urothelial cancer cells

**Figure 1:**
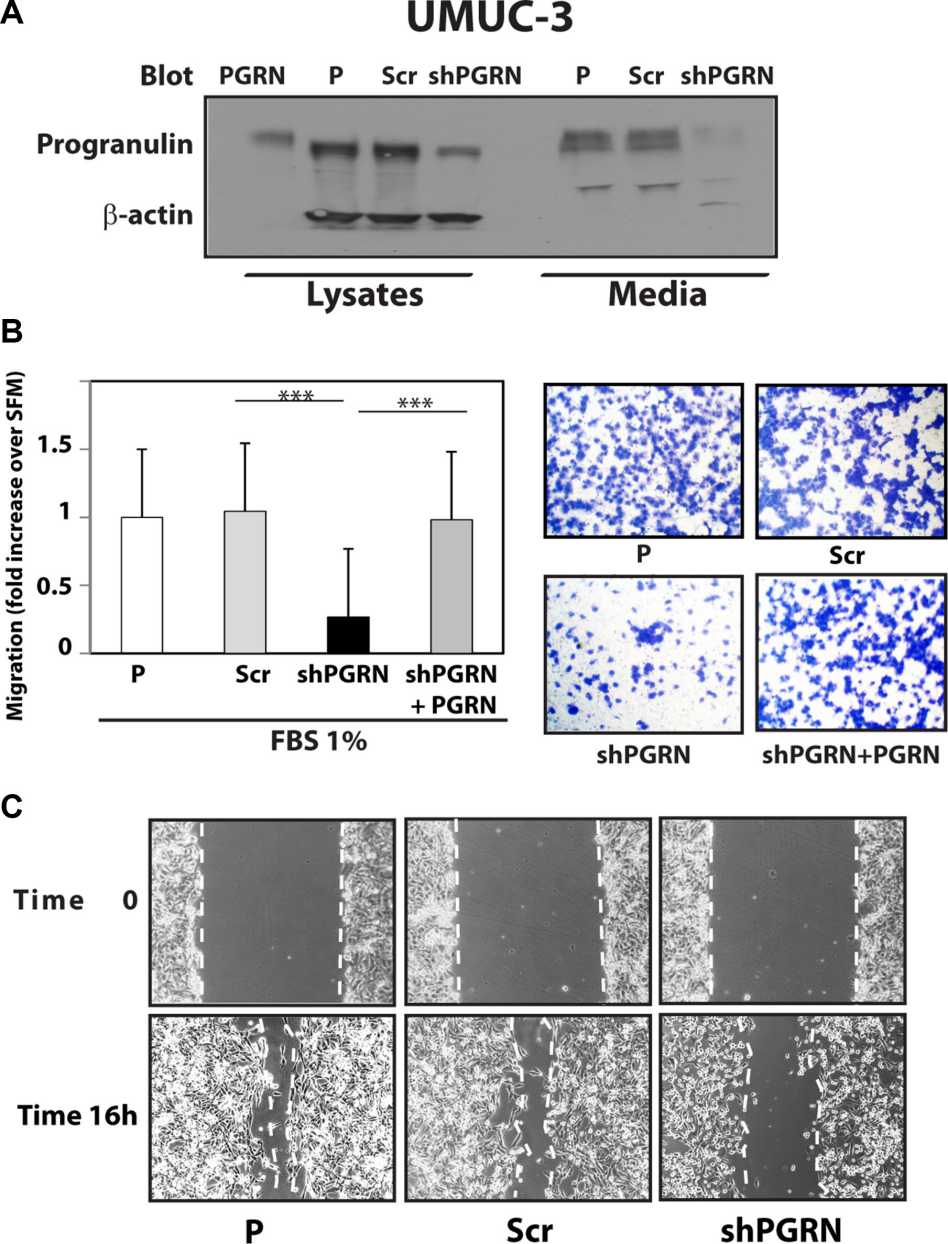
Progranulin depletion inhibits UMUC-3 urothelial cancer cell motility (**A**) The generation of UMUC-3/shScr (scramble control) and UMUC-3/shPGRN cells has been described in Materials and Methods. The progranulin-specific shRNA was TI350373 (Origine). Progranulin expression in lysates and conditioned media from parental (P), control (Scr) or shRNA progranulin-transfected (shPGRN) UMUC-3 cells was detected by immunoblot with anti-progranulin polyclonal antibodies. (**B**) Migration of the various UMUC-3 cell lines was performed using transwells as described in detail in Materials and Methods. Data are the average of three independent experiments run in duplicates ± SD. ***P < 0.001. Recombinant human progranulin was supplemented at 80 nM (**C**) The *in vitro* “wound healing” motility assay in UMUC-3 cells in either SFM or SFM supplemented with 1% FBS was performed as described in Materials and methods. Cells were analyzed with a cell live microscope using the Metamorph Image Acquisition and Analysis software (Universal Imaging) (×100). 10 fields/plate were examined. Pictures from representative fields are shown.

**Figure 2:**
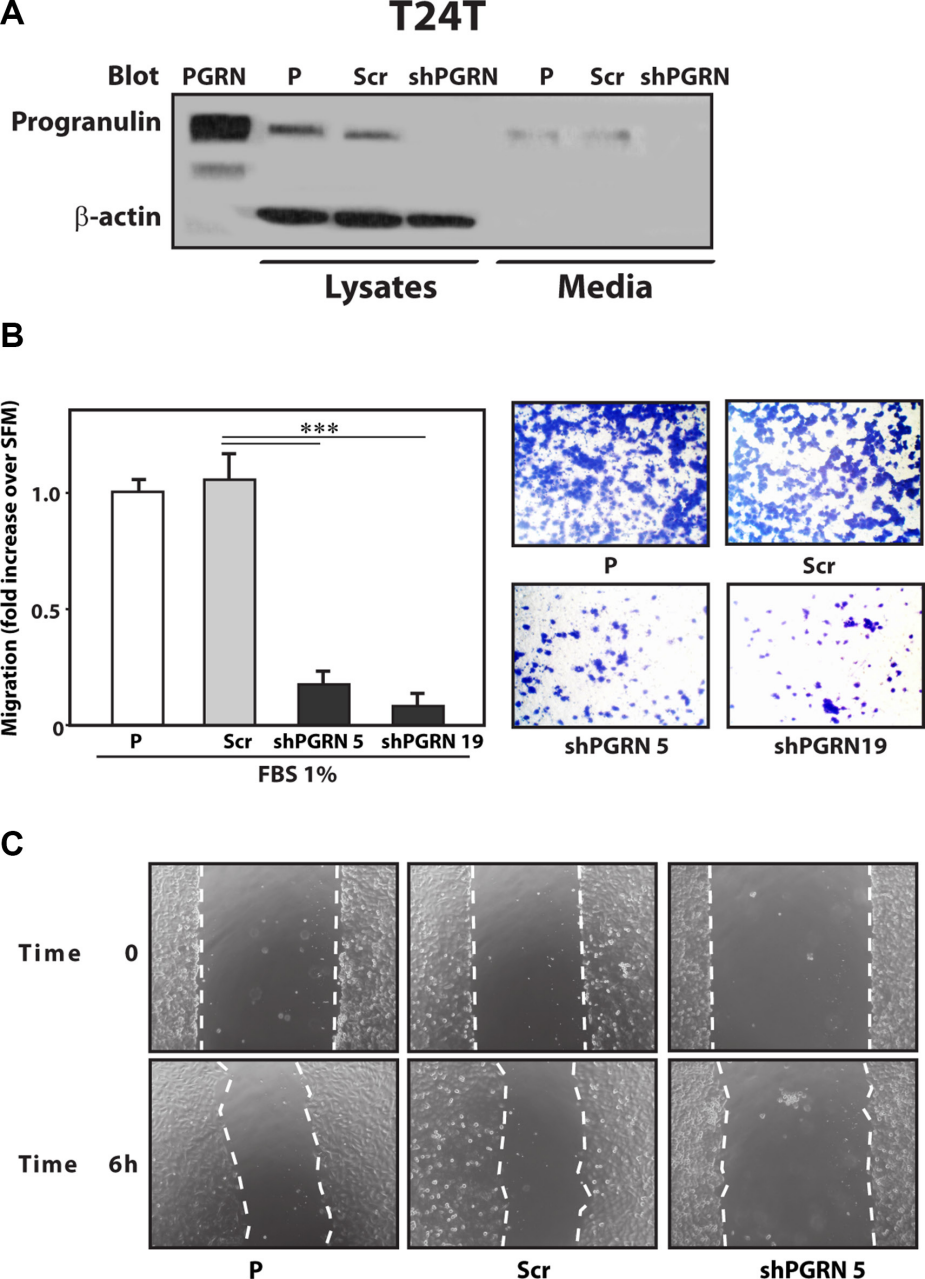
Progranulin depletion inhibits T24T urothelial cancer cell motility (**A**) The generation of T24T/shScr (scramble control) and T24T/shPGRN cells has been described in Materials and Methods. The progranulin-specific shRNA was TI350373 (Origine). Progranulin expression in lysates and conditioned media from parental (P), control (Scr) or shRNA progranulin-transfected (shPGRN) T24T cells was detected by immunoblot with anti-progranulin polyclonal antibodies. (**B**) Migration of the various T24T cell lines was performed using transwells as described in detail in Materials and Methods. Data are the average of three independent experiments run in duplicates ± SD. ***P < 0.001. (**C**) The *in vitro* “wound healing” motility assay in T24T cells in either SFM or SFM supplemented with 1% FBS was performed as described in Materials and methods. Cells were analyzed with a cell live microscope using the Metamorph Image Acquisition and Analysis software (Universal Imaging) (×100). 10 fields/plate were examined. Pictures from representative fields are shown.

Given the critical role of progranulin in regulating motility and invasion of urothelial cancer cells [15, 18, 19], we stably depleted endogenous progranulin in UMUC-3 and T24T urothelial cancer cells by transfecting expression plasmids expressing either a scrambled shRNA as control or a progranulin-specific shRNA. After selection, pools of UMUC-3 and T24T-transfected cells were tested by immunoblot for progranulin expression in both lysates and conditioned media [15, 18, 19]. The levels of progranulin secretion in media conditioned by UMUC- 3 (Figure 1A) or T24T (Figure 2A) transfected with the shPGRN plasmid were significantly (95%) reduced in both cell lines compared to parental (P) or scrambled-(Scr)-transfected cells. Progranulin depletion caused a robust inhibition (***P < 0.001) of the ability of UMUC- 3 (Figure 1B) and T24T (Figure 2B) cells to migrate. Importantly, motility was fully restored in UMUC-3/shPGRN by stimulation with nanomolar concentrations (~80 nM) of human recombinant progranulin (***P < 0.001, Figure 1B), thereby confirming that the inability of UMUC-3/shPGRN cells to migrate was due to progranulin ablation. Progranulin-depleted UMUC-3 (Figure 1C) and T24T (Figure 2C) cells were also considerably inhibited in their ability to close a wound as assessed by a wound healing lateral motility assay [15, 18, 19]. It is important to mention that we previously demonstrated that the ability of progranulin to promote lateral motility (wound healing) can be separated from the capacity to induce cell proliferation as in fact we previously determined at the wound site similar levels of BrdU incorporation between motile and cells unable to fill the wound [19], ruling out that progranulin depletion may affect wound healing just by affecting cell proliferation. In addition, wound healing was assessed at either 6 or 16 hours when cell proliferation would not be a major contributing factor.

### Progranulin depletion inhibits invasion and anchorage-independent growth

**Figure 3:**
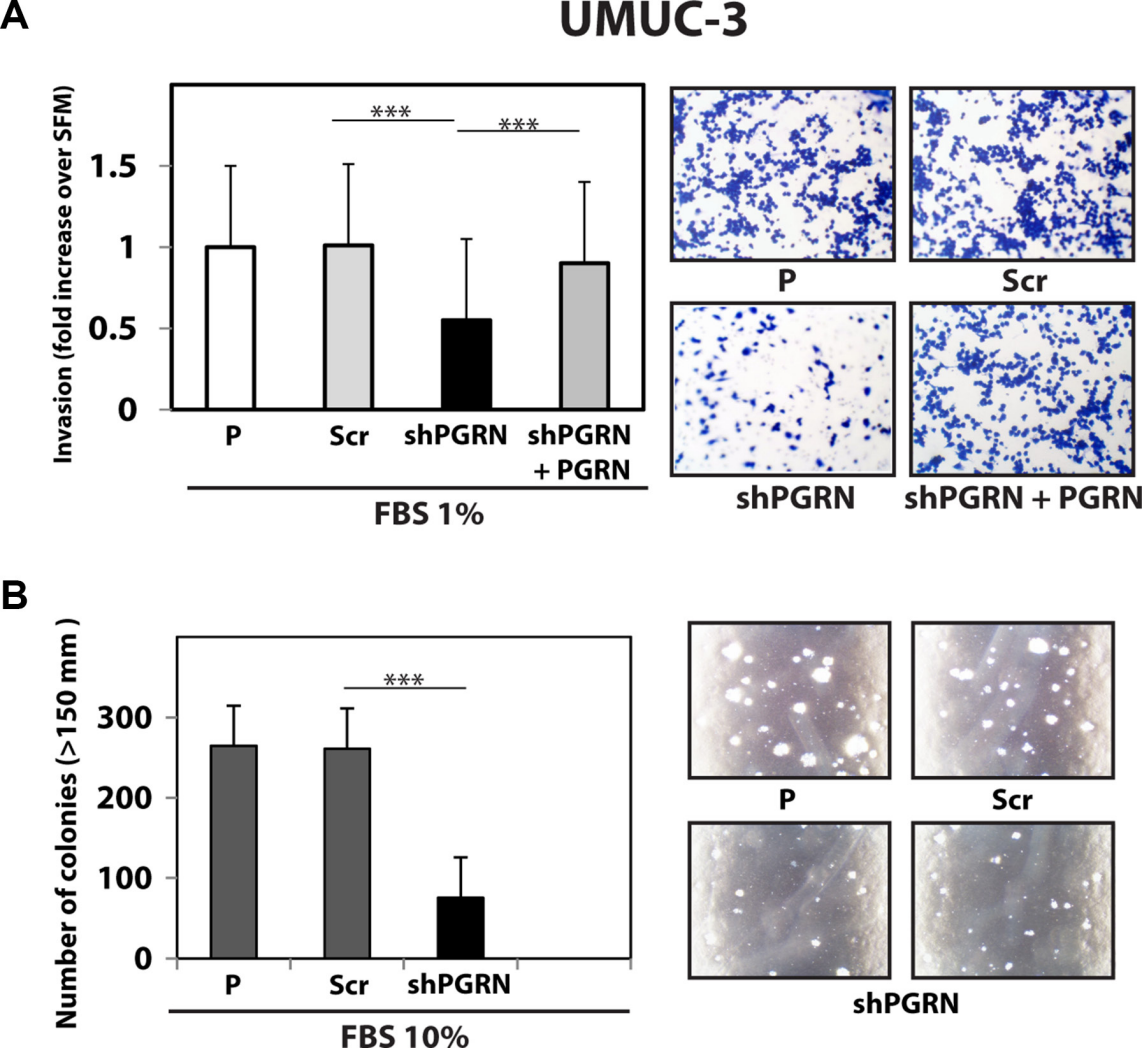
Progranulin targeting modulates invasion and anchorage-independent growth of UMUC-3 urothelial cancer cells (**A**) Parental (P), shScr-transfected (Scr) control and Progranulin-depleted (shPGRN) UMUC-3 cells were assessed for invasive ability through Matrigel-coated transwells as described in Materials and Methods. Data are the average of three independent experiments ± SD. ***P < 0.001. Recombinant human progranulin was supplemented at 80 nM. (**B**) Anchorage-independent growth was measured by colony formation in soft-agar as previously described [18, 19, 49]. Colonies > 150 μM were counted. The experiment is the average of three independent experiments run in duplicates ± SD. ***P < 0.001.

**Figure 4:**
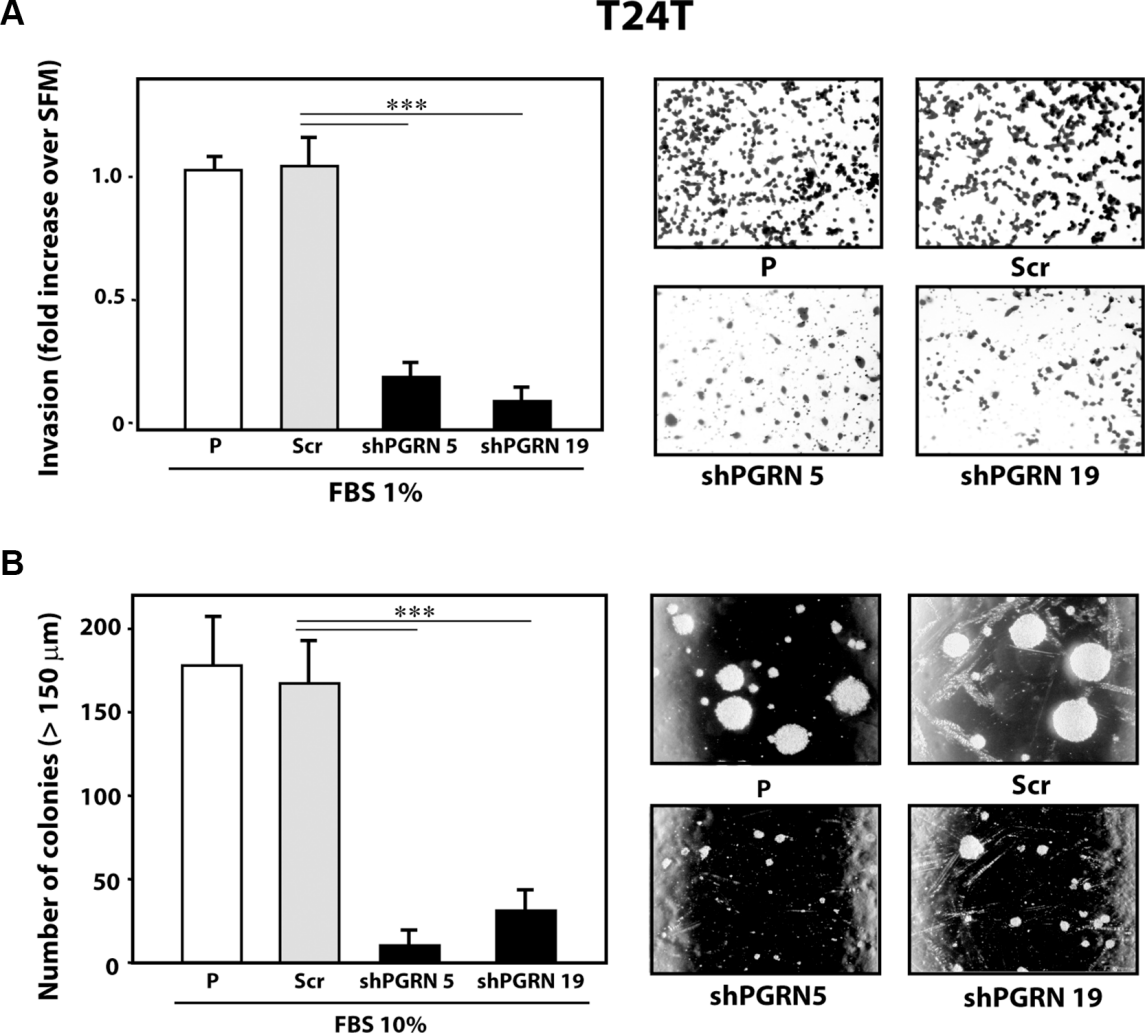
Progranulin targeting modulates invasion and anchorage-independent growth of T24T urothelial cancer cells (**A**) Parental (P), shScr-transfected (Scr) control and Progranulin-depleted (shPGRN) T24T cells were assessed for invasive ability through Matrigel-coated transwells as described in Materials and Methods. Data are the average of three independent experiments ± SD. ***P < 0.001. (**B**) Anchorage-independent growth was measured by colony formation in soft-agar as described IN Materials and Methods. Colonies >150 μM were counted. The experiment is the average of three independent experiments run in duplicates ± SD. ***P < 0.001.

Next, given the pro-motility activity of progranulin [15, 18, 19], we tested whether progranulin depletion would also affect the ability of urothelial cancer cells to invade through a 3D extracellular matrix. Thus, we used Matrigel-coated filters to examine invasive migration of progranulin-depleted UMUC-3 and T24T cells. Progranulin depletion significantly (***P < 0.001) affected the capacity of UMUC-3/shPGRN (Figure 3A) and T24T/shPGRN (Figure 4A) to invade as compared to parental and scrambled-transfected cells. Similarly to the migration assays, the invasive ability of UMUC- 3/shPGRN cells was fully restored (***P < 0.001) by recombinant human progranulin (Figure 3A). Notably, both UMUC-3/shPGRN (Figure 3B) and T24T/shPGRN cells (Figure 4B) were significantly impaired in forming colonies in a soft-agar assay (***P < 0.001).

**Figure 5:**
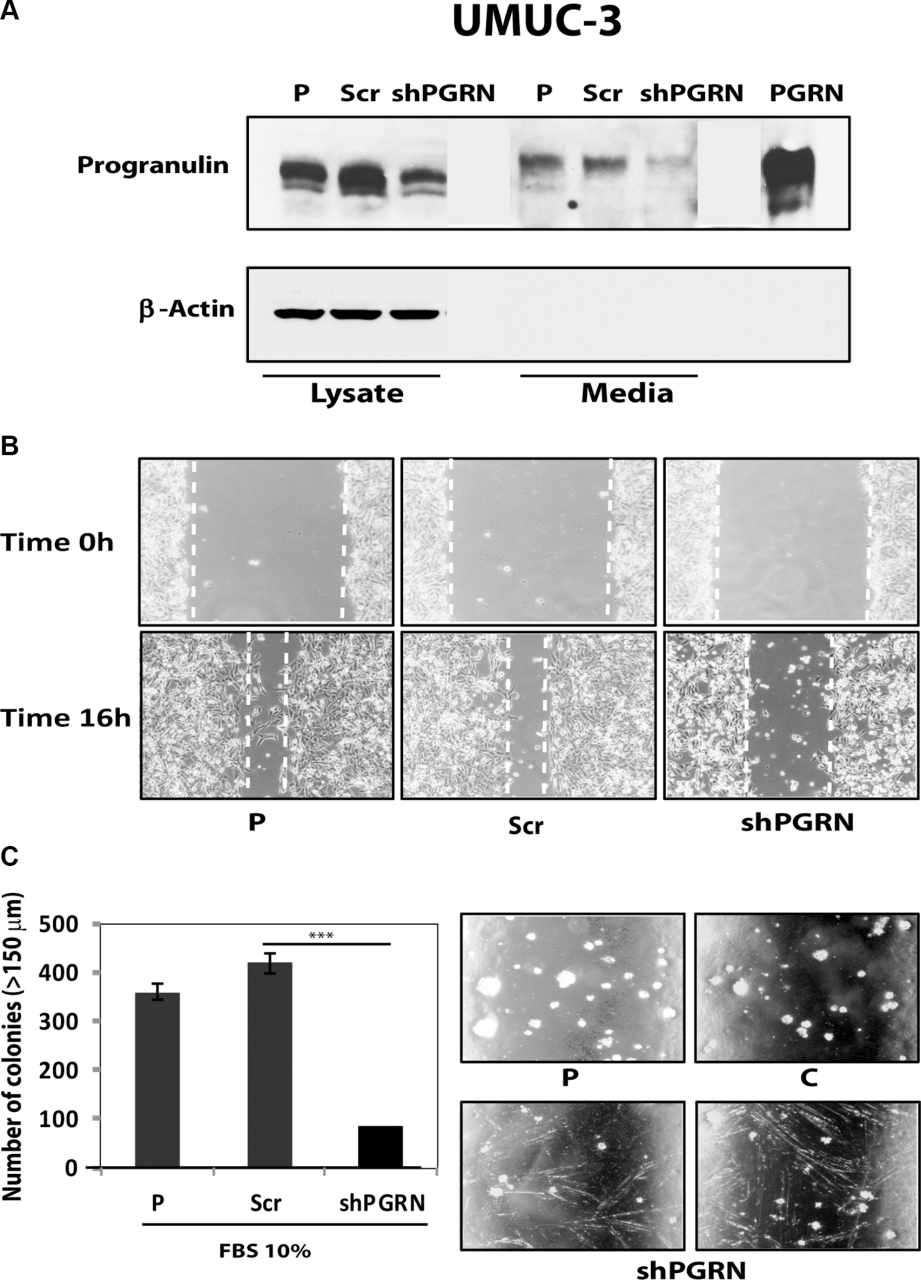
Progranulin depletion inhibits UMUC-3 urothelial cancer cell motility and anchorage-independent growth (**A**) The generation of UMUC-3/shScr (scramble control) and UMUC-3/shPGRN cells has been described in Materials and Methods. The progranulin-specific shRNA used here was TI350374 (Origine). Progranulin expression in lysates and conditioned media from parental (P), control (Scr) or shRNA progranulin-transfected (shPGRN) UMUC-3 cells was detected by immunoblot with anti-progranulin polyclonal antibodies. (**B**) The *in vitro* “wound healing” motility assay in UMUC-3 cells in either SFM or SFM supplemented with 1% FBS was performed as described in Materials and Methods. Cells were analyzed with a cell live microscope using the Metamorph Image Acquisition and Analysis software (Universal Imaging) (×100). 10 fields/plate were examined. Pictures from representative fields are shown. (**C**) Anchorage-independent growth was measured by colony formation in soft-agar as described in Materials and Methods. Colonies > 150 μM were counted. The experiment is the average of three independent experiments run in duplicates ± SD. ***P < 0.001.

As additional control, we generated progranulin-depleted UMUC-3 cells by transfecting a second progranulin-specific shRNA plasmids and isolated another pool of UMUC-3 cells with considerable reduction of progranulin production (Figure 5A). These cells also exhibited reduced motility (Figure 5B) and significant inhibition (***P < 0.001) of anchorage-independent growth (Figure 5C).

Collectively, these results indicate that progranulin expression levels exert an important role in regulating motility, invasion and anchorage-independent growth of bladder cancer cells.

### Progranulin modulates *in vivo* tumor formation

**Figure 6:**
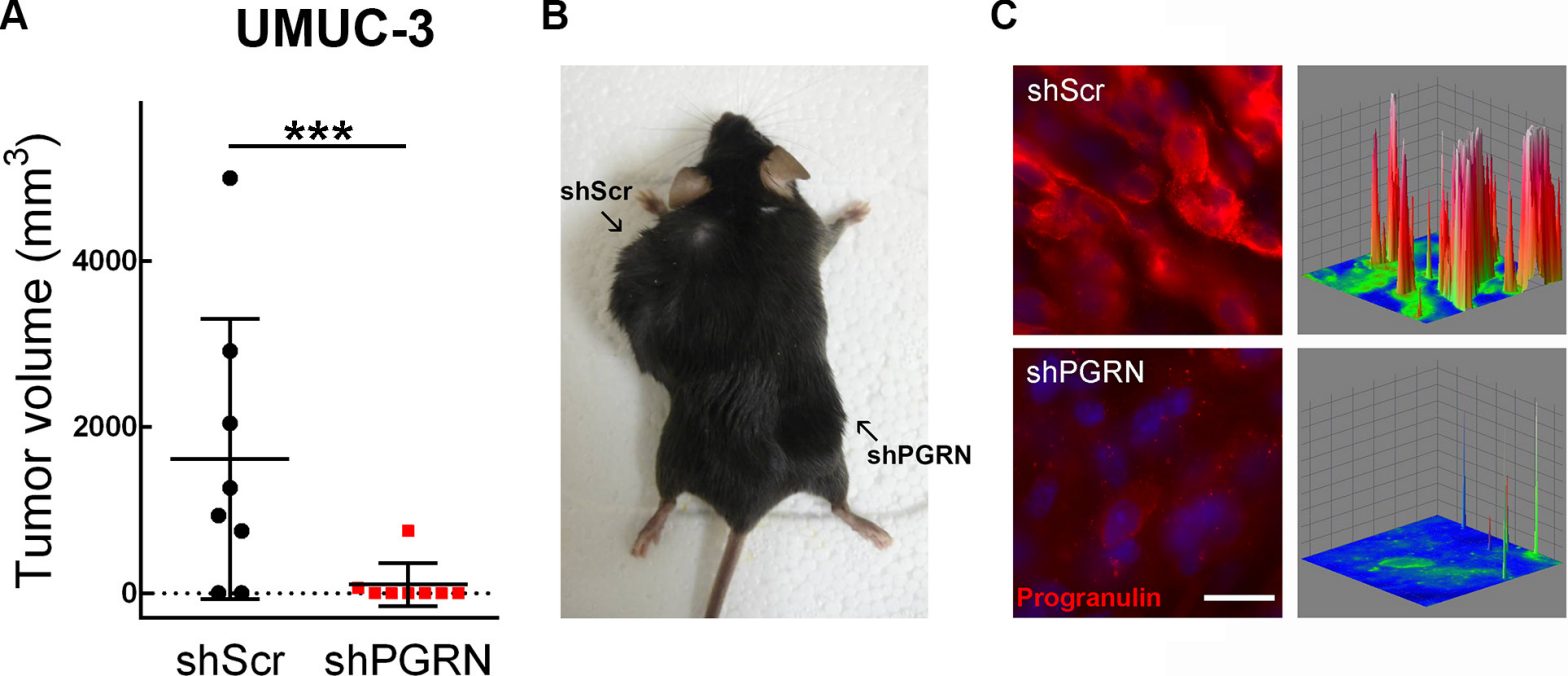
Stable depletion of endogenous progranulin in UMUC-3 urothelial cancer cells inhibits tumor xenograft growth (**A**) Xenograft volume at day 52 of mice injected with UMUC-3/shScr (shScr) and UMUC-3/shPGRN (shPGRN). ***P < 0.001. (**B**) Representative macroscopic photograph of a mouse 53 days post injection with UMUC-3/shScr in the left flank and UMUC3-shPGRN cell in the right flank. (**C**) Tumors extracted from UMUC-3/shScr and UMUC-3/sh-PGRN injected mice were tested for progranulin expression by immunofluorescence analysis. Three-dimensional surface plots are indicative of progranulin expression levels of the relative fluorescence images. Bar = 5 μm.

To extend the above *in vitro* finding into an *in vivo* setting, we generated tumor xenograft models and determined whether targeting progranulin could suppress the ability of UMUC-3 cells to form tumors. UMUC-3/shScr control and UMUC-3/shPGRN cells were implanted subcutaneously into the left and right flanks respectively of 6 week-old *Rag2*^−/−^ mice which lack the ability to initiate V(D)J rearrangement, thus severely immunocompromised. Once tumors were established, tumor sizes were monitored until the largest tumor reached ~5000 mm^3^ in size. Importantly, 70% of UMUC-3/shScr cells generated tumor xenografts (Figure 6A, 6B) in contrast to progranulin-depleted UMUC-3 cells of which only 12% generated xenografts with an average tumor volume significantly smaller than control (***P < 0.001, Figure 6A, 6B). To confirm that the tumor xenografts had reduced expression of progranulin, we performed immunofluorescence analysis on frozen sections of tumors after the animals were sacrificed. Indeed, progranulin levels were significantly depleted in the UMUC-3/shPGRN xenografts (Figure 6C) compared to UMUC-3/shScr control cells (Figure 6C).

**Figure 7:**
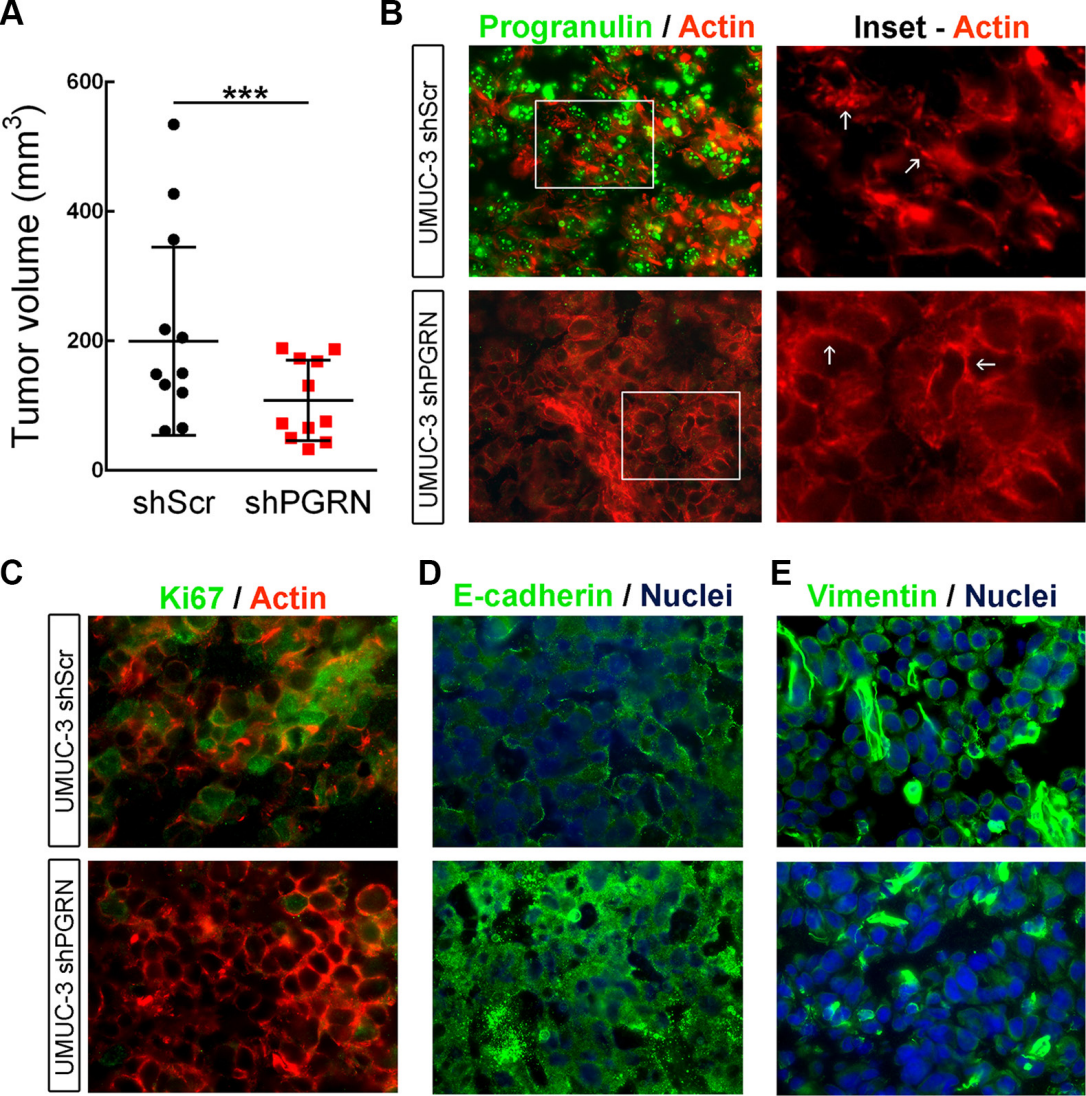
Progranulin regulates orthotopic bladder tumor formation (**A**) Tumor volume at day 20 of mice (*n* = 22) injected orthotopically, under ultrasound guidance, with UMUC-3/shScr (shScr) and UMUC-3/shPGRN (shPGRN) cells. ***P < 0.001. (**B**) Immunofluorescence staining of progranulin (green signal) and actin (red signal) in frozen sections of UMUC-3/shScr and UMUC-3/shPGRN orthotopic xenografts. The two insets represent relative magnified areas of the immunofluorescence pictures. White arrows point at the cortical actin fibers, which are disrupted in the presence of progranulin in the ShScr orthotopic tumors. (**C**–**E**) Immunostaining of Ki67, actin, E-cadherin and vimentin in frozen sections of the orthotopic tumors. Nuclei: blue. Bar = 10 μm.

To further expand the above findings regarding the role of progranulin in bladder tumor formation *in vivo*, we generated orthotopic bladder cancer xenografts by injecting UMUC-3/shScr and UMUC-3/shPGRN into the bladder of immunocompromised mice under ultrasound guidance. We then assessed growth as previously described [20, 21]. After 20 days, tumors derived from UMUC-3/shScr control cells reached an average of 200 mm^3^ of tumor volume (Figure 7A); in contrast, the progranulin depleted UMUC-3/shPGRN showed a significantly reduced tumor growth (***P < 0.001, Figure 7A). Given the marked inhibition in tumor growth of the progranulin-depleted tumors, we checked by immunofluorescence the levels of the protein Ki67, a cellular marker strictly associated with proliferation, in frozen sections of the orthotopic bladder cancer xenografts. Ki67 was significantly reduced in UMUC-3/shPGRN xenografts (10% positive nuclei) compared to UMUC-3/shScr tissue control (47% positive nuclei) (Figure 7C) suggesting that progranulin depletion inhibits tumor proliferation *in vivo*.

It is known that cancer cell motility and invasion require a change in cellular morphology associated with actin remodeling. Moreover, we have recently shown that exogenous stimulation of urothelial cancer cells with progranulin severely compromised the F-actin network of the cells [18]. Therefore, we examined the actin network of UMUC-3 orthotopic xenografts by staining frozen sections of the tumor tissue with the phallotoxin rhodamine-phalloidin. Notably, in UMUC-3/shPGRN derived tissue we could notice an intact and well assembled F-actin network, which was severely disrupted in control tissue derived from UMUC-3/shScr xenografts (Figure 7B, arrows in Insets), confirming our previous results indicating that progranulin regulates invasion, motility and proliferation of cancer cells by modulating F-actin remodeling [18]. As a further control of the suppression of progranulin in our tumor xenograft model, we tested the levels of progranulin by immunofluorescence analysis in the tumor tissue. Indeed, progranulin immunostaining resulted markedly reduced in frozen sections of UMUC-3/ShPGRN orthotopic xenografts compared to control tumor tissue (Figure 7B). Significantly, tumor tissues derived from UMUC-3/shScr xenografts showed reduced expression of the epithelial marker E-cadherin (Figure 7D) and enhanced levels of the mesenchimal protein vimentin (Figure 7E) compared to UMUC-3/shPGRN orthotopic xenografts suggesting that progranulin-associated tumorigenesis of UMUC-3 cells might be, at least in part, associated with an epithelial-to-mesenchimal (EMT) transition [22], which is severely affected by progranulin depletion.

Collectively, these findings provide a strong evidence for an important role for progranulin in regulating bladder tumor formation *in vivo*.

### Progranulin depletion sensitizes UMUC-3 cells to cisplatin treatment

**Figure 8:**
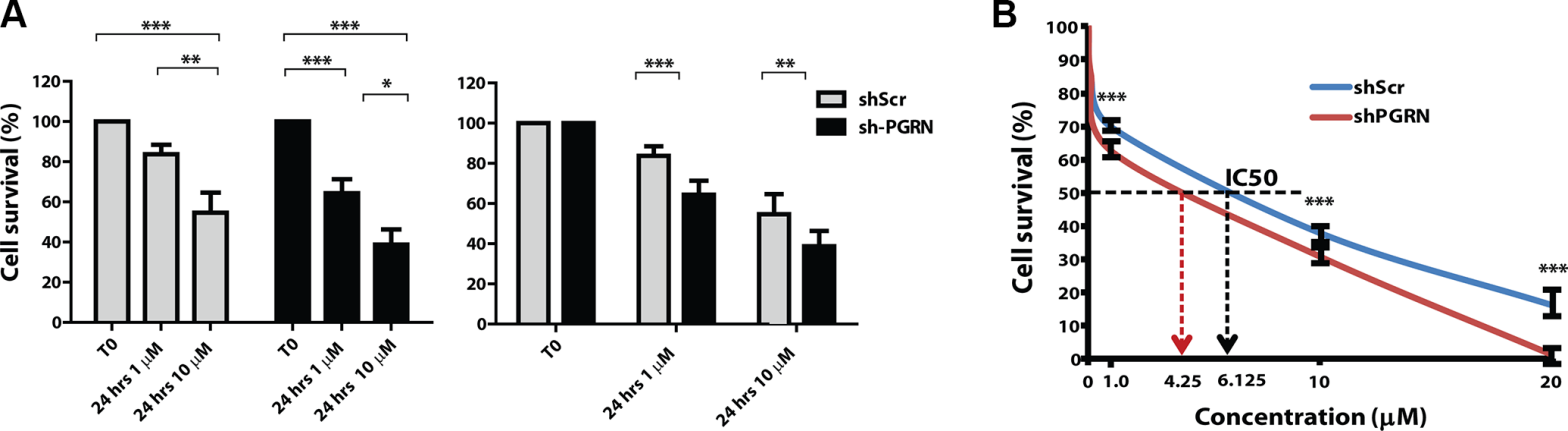
Progranulin depletion sensitized UMUC-3 urothelial cancer cells to cisplatin treatment (**A–B**) UMUC-3/shScr and UMUC-3/shPGRN were plated onto six-well plates at a density of 3.5 × 10^5^ cells/well in 10% FBS-containing media. After 24 hours, cells were washed and transfer to 1% FBS-containing media with or without cisplatin at the concentrations of 1 μM and 10 μM. Cells were then counted after 24 hours as previously described [31, 49, 50]. Data are the average of 5 independent experiments run in duplicates ± SD. Groups were compared by ANOVA. *P < 0.05. **P < 0.01. ***P < 0.001. (B) Cell survival was assessed in 1% serum-containing media by MTS assays at cisplatin concentration of 1, 10 and 20 μM. ***P < 0.001.

One of the standard treatments for invasive bladder cancer is based on utilizating platinum-containing anti-cancer drugs, such as cisplatin [23]. This chemotherapeutic agent binds to and causes cross-linking of DNA, which ultimately triggers DNA damage and apoptosis. Thus, we tested whether progranulin could contribute to regulate the sensitivity of invasive urothelial cancer cells to cisplatin treatment. UMUC-3/shScr and UMUC-3/shPGRN cells were grown in 1% serum supplemented with 1 or 10 μM cisplatin. Suppression of progranulin expression significantly enhanced cisplatin-dependent cell death at both 1 (***P < 0.001, Figure 8A) and 10 μM (**P < 0.05, Figure 8A), as compared to controls (Figure 8A). We confirmed these results performing MTS assays in the presence of increasing concentrations of cisplatin. UMUC- 3/shPGRN cells showed an IC_50_ of 4.25 μM compared to UMUC-3/shScr cells, which had an IC_50_ of 6.125 μM (Figure 8B). In addition cell survival was significantly decreased in UMUC-3/shPGRN compared to UMUC-3/shScr cells (***P < 0.001, Figure 8B) at all concentrations tested.

These results further strengthen the hypothesis that progranulin is a leading pro-survival factor in bladder cancer and suggest that targeting progranulin together with cisplatin could enhance therapeutic efficacy of cisplatin-based therapy in invasive bladder tumors.

### Progranulin expression in bladder cancer tissues

**Figure 9:**
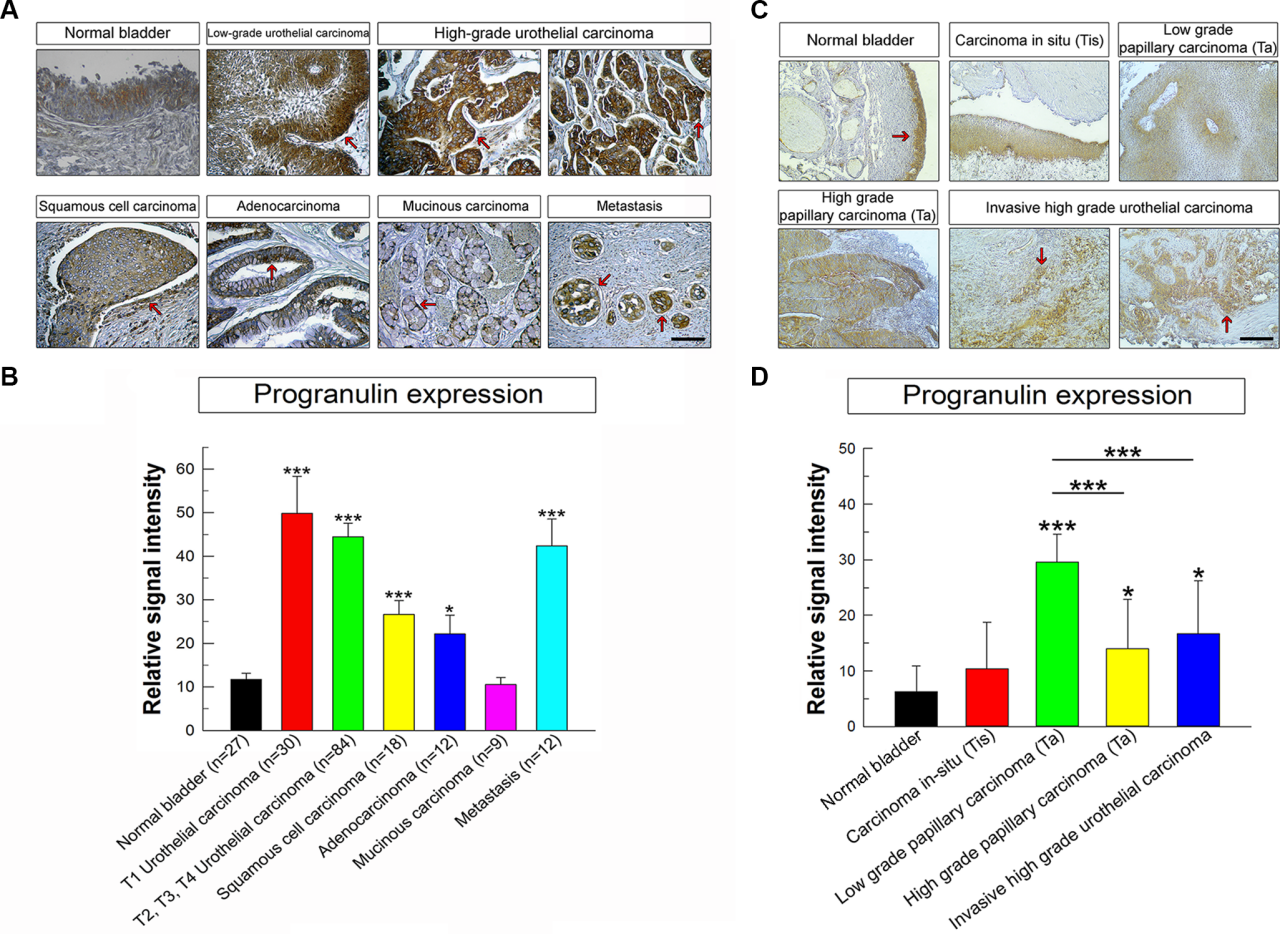
Progranulin is upregulated in bladder cancer tissues (**A**) Human bladder tumor microarray containing 69 validated cases of various types and stages of bladder cancers and normal bladder tissue controls. The panels show light micrographs outlining the expression of progranulin in different tumors as indicated. Progranulin is expressed at high levels in all cancers analyzed compared to the normal bladder except for the mucinous carcinoma. The arrows show areas of high staining for progranulin in the epithelial compartments of all cancer studied. (**B**) Quantification of progranulin staining levels was assessed using ImageJ software. All cases in the tissue microarray were analyzed and quantified. Briefly, the threshold of each image was adjusted in order to show only the specific staining. The background was removed and the data were quantified and plotted using SigmaPlot software. Student's *t*-tests were run to determine statistical significance of each class of cancers compared to normal bladder. *P < 0.05; ***P < 0.001. Bars = 100 μm. (**C**) Additional progranulin IHC on archived bladder cancer tissue samples, which include low and high grade superficial papillary tumors (Ta) and CIS (Ti). These results complement the TMA analysis with a broader spectrum of various urothelial carcinoma stages (**D**) Progranulin expression in archived tissue samples was analyzed and quantified as described above. *P < 0.05; ***P < 0.001.

Next, we assessed progranulin expression in human bladder cancer and determined whether these changes could be a prognostic factor for tumor progression. We previously showed in a very limited number of urothelial carcinoma cases (*n* = 5) that progranulin levels were upregulated in invasive tumors [15]. To expand these findings, we utilized a human bladder tumor microarray containing 69 validated cases, including various types and different stages of bladder cancers. In normal bladder, progranulin was barely detectable (Figure 9A, 9B). In contrast, all malignant bladder tumors exhibited a markedly-increased progranulin expression (***P < 0.001, Figure 9A, 9B). The only exception was mucinous carcinoma where progranulin expression was at levels comparable to normal tissue. Progranulin upregulation was not statistically different between T1 and T2-4 urothelial carcinoma tissues. Progranulin was also overexpressed in metastatic bladder tissues indicating that progranulin expression levels might be associated to bladder cancer metastasis (***P < 0.001, Figure 9A, 9B). In order to include in this analysis low grade superficial urothelial carcinomas, we tested progranulin expression levels in archived paraffin-embedded tissues derived from 4 non-invasive carcinoma *in-situ* (Tis), 4 non-invasive low grade papillary carcinomas (Ta), 4 non-invasive high grade papillary carcinomas (Ta) using as control normal and invasive high grade carcinoma tissues (*n* = 4). Progranulin expression levels were considerably up-regulated in non-invasive low grade papillary carcinomas (Ta) (***P < 0.001 compared to benign tissues, Figure 9C, 9D) and enhanced in both non-invasive high grade papillary carcinomas (Ta) and high grade carcinoma tissues (*P < 0.01 compared to benign tissues, Figure 9C, 9D). while in non-invasive carcinoma *in-situ* (Tis) progranulin expression was at levels comparable to benign tissues (Figure C, D).

## DISCUSSION

Muscle-invasive bladder tumors are biologically heterogeneous with variable clinical progression and response to chemotherapy [24]. Radical cystectomy with perioperative cisplatin-based chemotherapy is the standard of care for muscle-invasive bladder cancers [24] but no substantial advances in therapy have been made since the introduction of cisplatin-based regimens [24]. Therefore, there is an urgent need to identify novel predictor of disease progression and novel therapeutic targets for invasive bladder cancer.

We have recently demonstrated that progranulin modulates as autocrine growth factor motility and invasion of urothelial cancer cells. In addition, progranulin is overexpressed in invasive bladder tumor tissues compared to normal control suggesting that progranulin may work as an important driver of bladder tumor progression.

In the present study, we stably depleted endogenous progranulin in tumorigenic UMUC-3 and T24T urothelial cancer cells by shRNA approaches and show that: a) Progranulin depletion severely inhibits urothelial cancer cell motility and invasion, which are restored by stimulation with recombinant progranulin. b) Progranulin targeting significantly diminished the ability of urothelial cancer cells to grow in anchorage-independency. c) Progranulin expression levels are critical for *in vivo* tumor growth. d) Progranulin depletion sensitizes UMUC-3 cells to cisplatin treatment. e) Progranulin is upregulated in various bladder cancer–derived tissues compared to normal tissue controls.

Despite the important role that progranulin plays in bladder cancer, the mechanisms regulating progranulin action are still very poorly characterized. In addition, very few proteins that regulate the early stages of progranulin signaling from the plasma membrane have been so far identified.

Previous studies have discovered proteins of ~130 kDa as putative progranulin receptors [25, 26] and more recently, sortilin, a single-pass Type I transmembrane protein of the Vps10 family, has been identified in neuronal cells as a novel progranulin-interacting protein using membrane binding with alkaline phosphatase-labeled progranulin [27]. However, sortilin acts as negative regulator of progranulin levels and signaling by targeting progranulin for lysosomal degradation [27]. Reduced progranulin levels are associated with frontotemporal dementia similarly to haploinsufficiency associated with progranulin gene mutations [28, 29] and targeted manipulation of the sortilin/progranulin axis rescues progranulin haploinsufficiency [30].

Accordingly, we have recently demonstrated that sortilin is expressed at very low levels in castration-resistant PC3 and DU145 prostate cancer cells [31]. Restoring sortilin expression in these cells severely reduces progranulin levels and inhibits motility, invasion, proliferation and anchorage-independent growth [31]. Significantly, these results are recapitulated by targeting endogenous progranulin in PC3 and DU145 cells.

Progranulin has been shown to bind TNF receptors 1 and 2 and diminish TNF-dependent activation of MAPK (ERKs, p38 and JNK) by altering the TNF/TNFR interaction [32]. These results therefore do not support a role of TNFRs as *bona fide* progranulin receptor. In addition, in the presence of CpG-ONDs progranulin proteolytic fragments are soluble cofactors for Toll-like receptor 9 (TLR9) and regulate innate immunity [33]. Furthermore, Tropomyosin 3 has been discovered as a novel progranulin-interacting protein in hepatocellular carcinoma cells [34], but the biological significance of this interaction remains to be elucidated.

We have recently characterized a novel interaction between progranulin and the F-actin-binding protein drebrin [16, 17] and shown that drebrin is required for progranulin-induced activation of the Akt/MAPK pathways, regulates F-actin remodeling thereby modulating motility, invasion and anchorage-independent growth of urothelial cancer cells [18]. In addition, drebrin regulates *in vivo* tumor growth and its expression is upregulated in several human bladders cancers, irrespective of their histopathology [18]. These results were here confirmed in orthotopic tumors where in UMUC-3/shPGRN-derived tissue we detected an intact and well-assembled F-actin network, which was severely disrupted in control tissue derived from UMUC-3/shScr xenografts.

However, as drebrin is not a trans-membrane protein [16–18], it is not likely the progranulin cell-surface receptor. Instead, we can speculate that drebrin might be recruited at early stages of progranulin internalization from the cell membrane as part of a signaling multi-protein complex, which would include the putative progranulin membrane receptor.

We have also analyzed tissues derived from orthotopic xenografts for markers of the epithelial-to-mesenchimal (EMT) transition, which is an integral part of the transforming process [22]. Significantly, tumor tissues derived from UMUC-3/shScr xenografts showed reduced expression of the epithelial marker E-cadherin and enhanced levels of the mesenchimal protein vimentin compared to UMUC-3/shPGRN orthotopic xenografts. These preliminary results would suggest that the role of progranulin in bladder cancer tumor formation might be, at least in part, associated with an EMT transition [22], which would be reversed by progranulin targeting in urothelial cancer cells. These results are consistent with very recent data demonstrating that in ovarian cancer progranulin promotes motility and invasion through EMT and the activation of cancer-associated fibroblasts [35].

Our results provide the first demonstration that progranulin targeting may work as a novel therapeutic approach in bladder cancer extending previous results from breast carcinoma [36] and hepatocellular carcinoma-derived cell lines [37], where progranulin targeting by either antisense strategies [36] or inhibitory antibodies [37] respectively inhibits tumorigenicity.

The mechanisms by which progranulin modulates response to cisplatin are currently unknown. However, recent data in glioblastoma have demonstrated a role of progranulin in promoting Temozolomide resistance through the regulation of DNA repair and tumor cell stemness [38]. Future experiments will be devoted to characterize the role that progranulin may play in modulating DNA damage/repair in urothelial cancer cells.

There is a great interest at the moment in the identification of novel biomarkers for prognosis and treatment selection of advanced bladder cancers [39–41]. Recent work has identified subtypes of muscle-invasive bladder tumors, basal, luminal and p53-like that resemble molecular subtypes of breast cancer [41–44]. The basal subtype shows more aggressive disease at presentation and is characterized by squamous differentiation. Significantly, progranulin levels are significantly upregulated in squamous cell carcinoma-derived tissues suggesting that progranulin levels may contribute with other markers [41–44] to the identification of different invasive subtypes of bladder tumors.

Progranulin expression levels are not statistically different in low grade versus high grade urothelial carcinoma tissues. However, it is important to mention that the TMA we analyzed did not contain superficial papillary tumors (Ta) but only T1 tumors, which are low grade and not muscle-invasive but have penetrated the basement membrane [2], suggesting that progranulin expression might identify low grade tumors likely to progress to the muscle-invasive stage of urothelial carcinoma. The results of the additional analysis of progranulin expression in archived bladder cancer tissues for the most part confirms the TMA data and suggest high progranulin expression levels in both superficial and invasive tumors. Progranulin levels are instead low in carcinoma-*in-situ* samples at levels comparable to normal tissues. Collectively, these results are somewhat surprising considering the role of progranulin in motility and invasion and indicate the progranulin levels are predictive of bladder tumor formation but do not separate superficial from invasive tumors. Because we have recently shown that drebrin is instead highly expressed in high grade invasive tumors compared to low grade superficial and normal controls [18], these results might indicate that progranulin downstream effectors, such as drebrin or the unidentified progranulin receptor, are likely better predictor of tumor progression than progranulin itself.

Notably, progranulin expression levels are also upregulated in metastatic urothelial carcinoma-derived tissues compared to normal bladder tissue controls. These results would support the hypothesis that progranulin by enhancing urothelial cancer cell motility and invasion may be critical not only for the early stages of the metastatic cascade, invasion from the primary site, intravasation and extravasation, but also important when migrated tumor cells reach the secondary site of metastasis, where progranulin might work as contributing factor to the colonization process [45]. Experiments aiming at the characterization of progranulin action in the metastatic colonization process are currently under way.

In summary, our studies have demonstrated a critical role for progranulin in regulating motility, invasion, anchorage-independent growth *in vitro* and tumor formation *in vivo* of urothelial cancer cells. In addition, progranulin sensitizes invasive bladder cancer cells to cisplatin treatment. Progranulin may constitute therefore a novel target for therapeutic intervention in bladder tumors.

## MATERIALS AND METHODS

### Cell lines

Urothelial carcinoma-derived human UMUC-3 cells were obtained by ATCC (Manassas, VA, USA). T24T cells (a kind gift from Dr. Dan Theodorescu, University of Colorado Cancer Center) are a more aggressive isogenic derivatives of T24 cells and grow in soft-agar, [46–48].

UMUC-3 cells were maintained in MEM with EARL medium with 10% fetal bovine serum (FBS), while T24T cells were maintained in DMEM/F12 medium supplemented with 10% fetal bovine serum (FBS). Serum-free medium (SFM) is DMEM supplemented with 0.1% bovine serum albumin and 50 μg/ml of transferrin (Sigma-Aldrich, St Louis, MO, USA).

### Generation of progranulin-depleted UMUC-3 cells

UMUC-3 and T24T cells stably depleted of endogenous progranulin were generated by transfecting the pRS/shScr (scrambled shRNA) and pRS/shPGRN plasmids (OriGene Technologies, Inc.) using the TransIT^®^-Prostate Transfection Kit (Mirus).

Sequences of the two progranulin-specific 29mer shRNA were:

TI350373: “GAGTAAGTGCCTCTCCAAGGAGAACGCTA”

TI350374: “GGAAGGACACTTCTGCCATGATAACCAGA”

Cells were selected in medium supplemented with 2 μg/ml of puromycin. After selection, pools of progranulin-depleted UMUC-3 and T24T cells were tested for progranulin expression levels in both lysates and conditioned media by immunoblot using anti-progranulin polyclonal antibodies (USBiologicals, Swampscott, MA, USA) as previously described [15, 31].

### Migration, wound healing and invasion assays

HTS FluoroBloks^TM^ inserts (Becton Dickinson, Durham, NC, USA) were saturated with PBS-1% bovine serum albumin for 2 hours at room temperature. Serum-starved cells were labeled with DiI (Molecular Probes, Grand Island, NY, USA) for 20 minutes at 37°C and then seeded in the HTS FluoroBloks^TM^ upper chamber in either SFM or SFM supplemented with 1% FBS and incubated at 37°C for 18 hours. After fixing in 4% paraformaldehyde, membranes were mounted on a slide and migrated cells were counted and photographed with a Zeiss Axiovert 200 M cell live microscope at the Kimmel Cancer Center Confocal Microscopy Core Facility.

Recombinant human progranulin was generated as previously described [8].

Wound healing was assessed in either SFM or SFM supplemented with 1% FBS by a scratch assay in monolayer has described in previous works from our laboratories [15, 31].

Cell invasion through a three-dimensional extracellular matrix was measured by a Matrigel invasion assay using BD Matrigel™ Invasion Chambers (BD Biocoat, Bedford, MA, USA) with 8.0 μm filter membranes. Cells (5 × 10^4^) in 200 μl of SFM were plated onto each filter, and 750 μl of SFM or SFM supplemented with 1% FBS in the lower chamber. After 24 hours filters were washed, fixed, and stained with Coomassie Brilliant Blue. Cells on the upper surface of the filters were removed with cotton swabs. Cells that had invaded to the lower surface of the filter were counted under the microscope.

### Colony formation assay in soft-agar

Colony formation in soft-agar was performed as previously described [18, 19, 31, 49]. Cells were seeded in soft-agar at a density of 5 × 10^3^ cells/35 mm plate and counted after three weeks in culture. Colonies > 150 μm were scored as positive.

### Bladder cancer xenografts

Experiments using 7- to 10-week-old *Rag*2−/− mice were carried out according to protocols approved by the Institutional Review Board of Thomas Jefferson University. 4 × 10^6^ UMUC-3/shScr (control) and shPGRN cells were injected subcutaneously in two distinct sites of mice flanks (control cells in the upper-left flank and progranulin-depleted cells in the lower-right flank). Three independent experiments with 6 mice each were performed. Once tumors were established, tumor growth was measured every 2 days with a micro-caliper utilizing the following formula: V = a(b^2^/2), where a and b represent the larger and small diameters, respectively. When the largest tumor reached 5000 mm^3^ in size, mice were sacrificed and tumors were surgically dissected and divided. One half of each tumor was snap frozen in liquid nitrogen for further biochemical analysis, whereas the other half was embedded in OCT medium (Sakura Finetek, Torrance, CA, USA) and frozen at −20°C. 8 μm thick cryostat sections were cut from the blocks, mounted on slides and subjected to immunofluorescence analysis utilizing a rabbit anti-progranulin antibody (Abcam, Cambridge, MA, USA). Three-dimensional surface plots were created with ImageJ and represent progranulin expression based on the intensity of the immunofluorescence signal.

### Orthotopic bladder cancer xenograft model

Orthotopic xenograft experiments were performed at the Vancouver Prostate Centre according to Canadian Council of Animal Care guidelines and approved by the Institutional Review Board of the University of British Columbia. Briefly, 22 nine weeks old female athymic nude mice (NU-Foxn1^nu^, Harlan Laboratories, Indianapolis, IN) with the average weight of 24.3 g were assigned into 2 groups by weight. Tumor inoculation was performed by ultrasound-guided injection according to the technique developed in our laboratories [20, 21]. Mice were anesthetized with 2.5% isoflurane plus meloxicam 1 mg/ Kg (Boehringer Ingelheim Vetmedica, St. Joseph, MO) subcutaneously. Animals were placed one by one in supine position on the heated imaging platform (Vevo770, small animal imaging system, VisualSonics, Toronto, ON). After swabbing the lower abdomen with chlorhexidine 0.5%, application of sterile ultrasound gel and visualization of the bladder on the screen, bladder cancer cell suspension (50 μL of Matrigel with 10^4^ cells, UMUC-3/shScr and UMUC-3/shPGRN) was injected into anterior bladder wall between its muscle layer and mucosa (30G ¾” needle and 1 mL syringe).

For *in vivo* tumor growth monitoring we used ultrasound 3D imaging and subsequent volume calculations starting on day 7 after tumor inoculation and then once weekly for 4 weeks total. Tumor take was 100%. All mice were euthanized (isoflurane anesthesia followed by CO_2_ asphyxiation) on day 26 post-inoculation when some tumor volumes reached 500 mm^3^ (10 mm in diameter). Bladders were then removed, drained from urine and weighed on analytical balance. Each tumor was divided in half; one part was snap frozen with liquid nitrogen and stored at −80°C and the other kept in 10% buffered formalin for 24 hours and then stored in 70% isopropyl alcohol. 8 μm thick cryostat sections were cut from the frozen tissue once embedded in OCT, mounted on slides and further processed for immunofluorescence analysis utilizing a rabbit anti-ki67 and a rabbit anti-granulin antibody (Abcam, Cambridge, MA, USA). Actin staining was obtained by incubation of the slides with rhodamine-phalloidin (Thermo Fisher, USA) according to the manufacturer's protocol. E-cadherin and vimentin expression was detected using specific rabbit polyclonal antibodies (Cell Signaling, MA, USA) following by staining with Alexa-Fluor secondary antibody (Thermo Fisher Scientific, MA, USA).

### Cell viability assay in the presence of cisplatin

UMUC-3/shScr and UMUC-3/shPGRN were plated onto six-well plates at a density of 3.5 × 10^5^ cells/well in media supplemented with 10% FBS. After 24 hours, cells were washed twice with PBS 1× and transfer to 1% FBS-supplemented media with or without cisplatin (Sigma-Aldrich, St Louis, MO, USA) at the concentrations of 1 μM and 10 μM. Cells were then counted after 24 hours in a boyden chamber.

Cell survival was additionally tested by MTS assays (Abcam) following the manufacturer's instructions.

### Immunohistochemical detection of progranulin expression in bladder cancer tissues

Progranulin expression was analyzed by immunohistochemistry on a Biomax (Rockville, MD, USA) human bladder cancer tissue microarray (BL208) at the Translational Core Facility of the Sidney Kimmel Cancer Center. The antibody used was an anti-granulin rabbit monoclonal antibody (Abcam, ab108608) at a dilution of 1:400. Detailed specifications of the bladder tissue array can be found at: http://www.biomax.us/tissue-arrays/Bladder/BL208.

Archived paraffin-embedded tissues from 4 non-invasive carcinoma *in-situ* (Tis), 4 non-invasive low grade papillary carcinomas (Ta), 4 non-invasive high grade papillary carcinomas (Ta) and control benign and invasive high grade carcinoma tissues (*n* = 4) were stained for progranulin expression as described above. All cases in the tissue microarray and from archived samples were analyzed for progranulin expression and quantified. Briefly, the threshold of each image was adjusted in order to show only progranulin-specific staining. The background was removed and the data were quantified and plotted using SigmaPlot software. Student's *t*-tests were run to determine statistical significance of each class of cancers compared to normal bladder.

### Statistical analysis

Results of multiple experiments are expressed as mean ± SD. All statistical analyses were carried out with SigmaStat for Windows version 3.10 (Systat Software, Inc., Port Richmond, CA). Results were compared using the two-sided Student's *t* test. Differences were considered statistically significant at *P* < 0.05.

## Abbreviations

GEP: granulin-epithelin precursor
PCDGF: PC-cell-derived growth factor
SFM: serum-free medium
BC: bladder cancer
