# Correction: Suppression of progranulin expression inhibits bladder cancer growth and sensitizes cancer cells to cisplatin

**DOI:** 10.18632/oncotarget.28657

**Published:** 2024-10-01

**Authors:** Simone Buraschi, Shi-Qiong Xu, Manuela Stefanello, Igor Moskalev, Alaide Morcavallo, Marco Genua, Ryuta Tanimoto, Ruth Birbe, Stephen C. Peiper, Leonard G. Gomella, Antonino Belfiore, Peter C. Black, Renato V. Iozzo, Andrea Morrione

**Affiliations:** ^1^Department of Pathology, Anatomy and Cell Biology and The Cancer Cell Biology and Signaling Program, Kimmel Cancer Center, Thomas Jefferson University, PA, Philadelphia, USA; ^2^Department of Urology and Biology and The Prostate Cancer Program, Kimmel Cancer Center, Thomas Jefferson University, PA, Philadelphia, USA; ^3^Vancouver Prostate Centre, Department of Urologic Sciences, University of British Columbia, Vancouver, Canada; ^4^Department of Health and Endocrinology, University Magna Graecia of Catanzaro, Catanzaro, Italy; ^*^These authors contributed equally to this work

**This article has been corrected:** During the preparation of the invasion data cell visualization box in Figure 3A, a partially overlapping parental (P) cell field instead of the Scr (Scramble control) cell field was mistakenly duplicated. The corrected Figure 3A, obtained using the original data, is shown below. The authors declare that these corrections do not change the results or conclusions of this paper.

Original article: Oncotarget. 2016; 7:39980–39995. 39980-39995. https://doi.org/10.18632/oncotarget.9556

**Figure 3:**
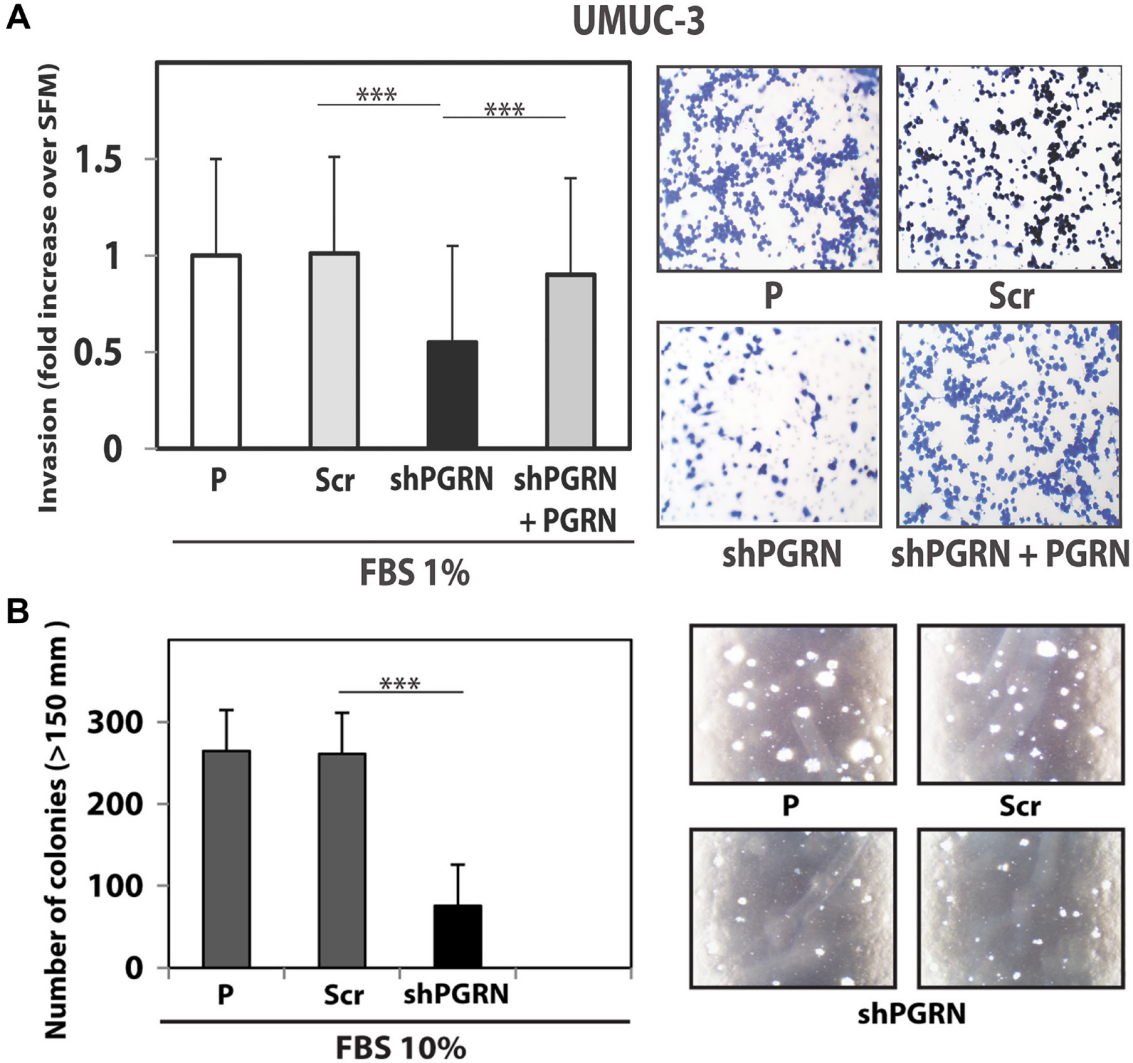
Progranulin targeting modulates invasion and anchorage-independent growth of UMUC-3 urothelial cancer cells. (**A**) Parental (P), shScr-transfected (Scr) control and Progranulin-depleted (shPGRN) UMUC-3 cells were assessed for invasive ability through Matrigel-coated transwells as described in Materials and Methods. Data are the average of three independent experiments ± SD. ^***^ *P* < 0.001. Recombinant human progranulin was supplemented at 80 nM. (**B**) Anchorage-independent growth was measured by colony formation in soft-agar as previously described [18, 19, 49]. Colonies > 150 μM were counted. The experiment is the average of three independent experiments run in duplicates ± SD. ^***^ *P* < 0.001.

